# Correction: PERK reprograms hematopoietic progenitor cells to direct tumor-promoting myelopoiesis in the spleen

**DOI:** 10.1084/jem.2021149805312022c

**Published:** 2022-06-08

**Authors:** Mingyu Liu, Chong Wu, Shufeng Luo, Qiaomin Hua, Hai-Tian Chen, Yulan Weng, Junyu Xu, Huiling Lin, Lu Wang, Jinheng Li, Lan Zhu, Zhenhong Guo, Shi-Mei Zhuang, Tiebang Kang, Limin Zheng

Vol. 219, No. 4 | 10.1084/jem.20211498 | March 10, 2022

The authors regret that in the original version of their article, the symbols and lines for the “Veh; host-derived” group were inadvertently swapped with those of the “Veh; donor-derived” group in Fig. 1 K during manuscript preparation. In Fig. 3 D, the symbols and lines for the “GSK → Hepa” group were inadvertently swapped with those of the “4µ8C → Hepa” group. The figure legends and article text remain unchanged. The corrected Figs. 1 and 3 are shown here.

**Figure 1.**
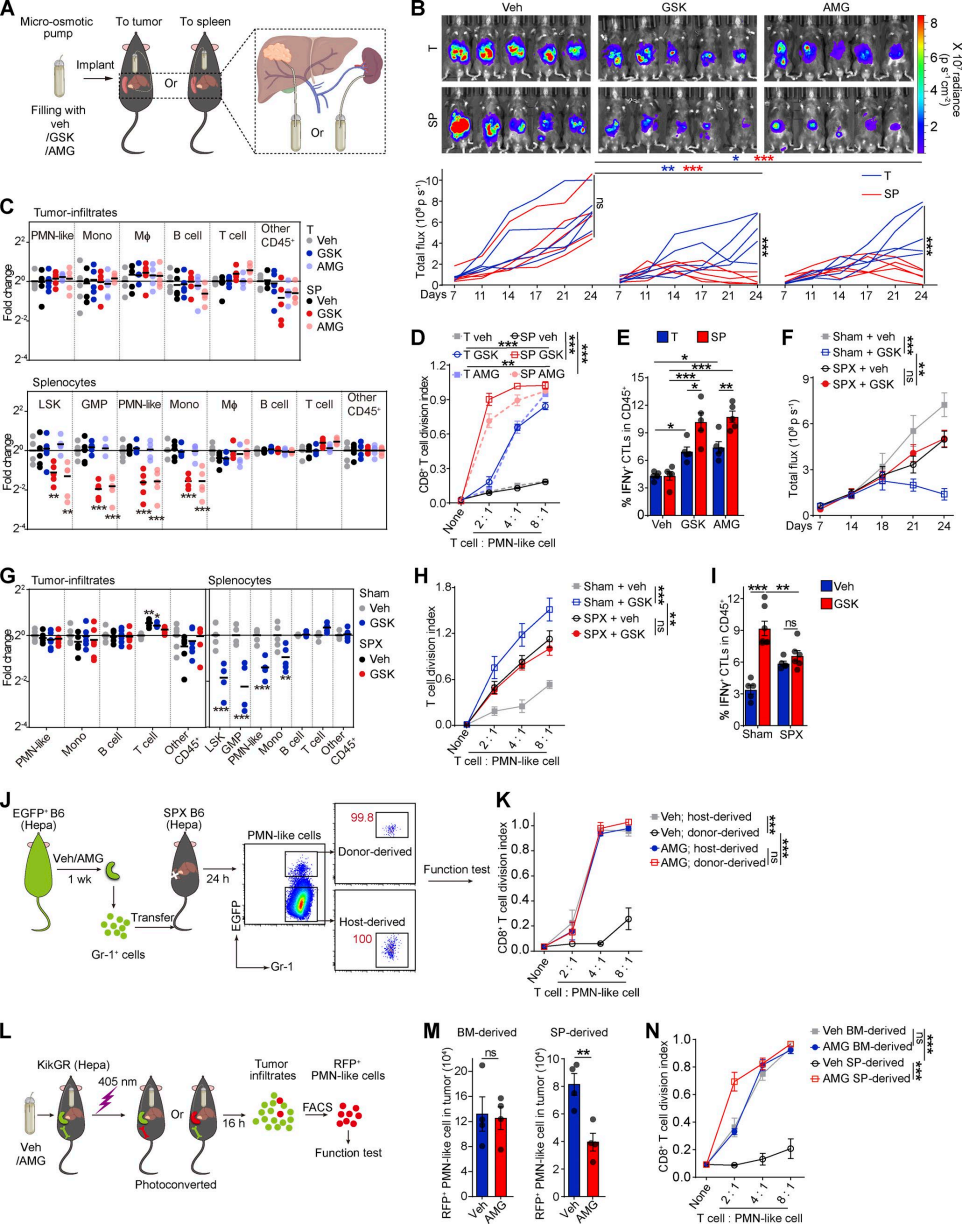


**Figure 3.**
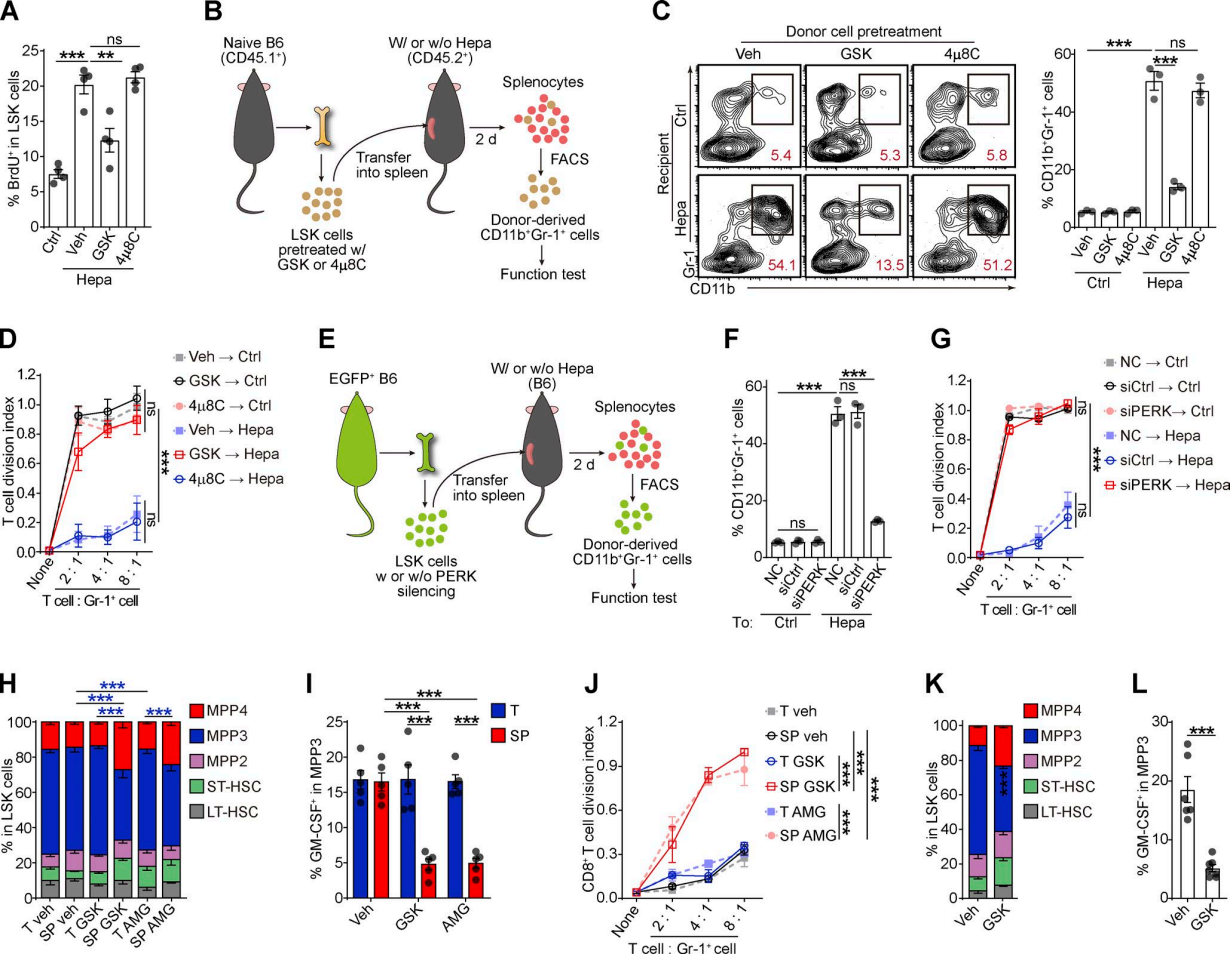


The errors appear in print and in PDFs downloaded before June 1, 2022.

